# Effect of liver dysfunction on outcome of radioactive iodine therapy for Graves’ disease

**DOI:** 10.1186/s12902-022-01242-w

**Published:** 2022-12-15

**Authors:** Yuyang Ze, Fei Shao, Xuefeng Feng, Shanmei Shen, Yan Bi, Dalong Zhu, Xiaowen Zhang

**Affiliations:** 1 Department of Endocrinology, Drum Tower Hospital affiliated to Nanjing University Medical School, Branch of National Clinical Research Centre for Metabolic Diseases; Endocrine and Metabolic Disease Medical Center, Drum Tower Hospital affiliated to Nanjing University Medical School, No. 321 Zhongshan Road, Nanjing, 210000 Jiangsu China; 2 Department of Endocrinology and Metabolism, the Fifth People’s Hospital of Suzhou Wujiang, No. 555, Xinyou Road, Suzhou, 215200 China; 3 Department of Endocrinology and Metabolism, Langxi Hospital of Traditional Chinese Medicine, No. 99 Tingzishan Road, Jianping Town, Langxi County, Xuancheng City, 242100 Anhui China; 4 Department of Nuclear Medicine, Drum Tower Hospital affiliated to Nanjing University Medical School, No. 321 Zhongshan Road, Nanjing, 210000 China

**Keywords:** Graves’ disease, Liver dysfunction, Radioactive iodine therapy (RAI), Radioactive iodine therapy uptake rate (RAIU), Thyroid ultrasound

## Abstract

Liver dysfunction is a common complication of Graves’ disease (GD) that may be caused by excessive thyroid hormone (TH) or anti-thyroid drugs (ATDs). Radioactive iodine (RAI) therapy is one of the first-line treatments for GD, but it is unclear whether it is safe and effective in patients with liver dysfunction. 510 consecutive patients with GD receiving first RAI were enrolled in the study, and followed up at 3-, 6- and 12-month. Liver dysfunction was recorded in 222 (43.5%) patients. GD patients with liver dysfunction had higher serum levels of free triiodothyronine (FT3) (median 27.6 vs. 20.6 pmol/L, *p* < 0.001) and free thyroxine (FT4) (median 65.4 vs. 53.5 pmol/L, *p* < 0.001) levels than those with normal liver function. Binary logistic regression analysis showed that duration of disease (OR = 0.951, 95% CI: 0.992–0.980, *p* = 0.001) and male gender (OR = 1.106, 95% CI: 1.116–2.384; *p* = 0.011) were significant differential factors for liver dysfunction. Serum TSH levels were higher in patients with liver dysfunction at all 3 follow-up time points (*p* = 0.014, 0.008, and 0.025 respectively). FT3 level was lower in patients with liver dysfunction at 3-month follow-up (*p* = 0.047), but the difference disappeared at 6 and 12 months (*p* = 0.351 and 0.264 respectively). The rate of euthyroidism or hypothyroidism was higher in patients with liver dysfunction than in those with normal liver function at 3 months (74.5% vs 62.5%; *p* = 0.005) and 6 months (82.1% vs 69.1%; *p* = 0.002) after RAI treatment, but the difference did not persist at 12-month follow-up (89.6% vs 83.2%, *p* = 0.081).There were no statistically significant differences in treatment efficacy (94.48% vs 90.31%, *p* = 0.142), incidence of early-onset hypothyroidism (87.73% vs 83.67%, *p* = 0.277), and recurrence rate (4.91% vs 7.14%, *p* = 0.379) between the 2 groups at 12-month follow-up. In conclusion, the efficacy of RAI was comparable in GD patients with liver dysfunction and those with normal liver function.

**Supplementary Information:**

The online version contains supplementary material available at 10.1186/s12902-022-01242-w.

## Introduction

Graves’ disease (GD) is the most common cause of hyperthyroidism, accounting for 50%–80% of cases [1]. The recommended treatments for patients with overt Graves’ hyperthyroidism include anti-thyroid drugs (ATDs), radioactive iodine (RAI) therapy, and thyroidectomy [2, 3]. RAI therapy remains the most frequently used treatment approach for patients with GD in the United States, while ATDs are preferred in Europe, Latin America, and Asia [4]. RAI therapy is particularly suitable for GD patients with a poor response to ATDs, patients with relapsed Graves’ hyperthyroidism, or those who have ATD-related adverse events such as hepatic dysfunction [2, 5].

Liver dysfunction is a common complication of GD that may be caused by excessive thyroid hormone, ATDs, or other types of liver disease. Although most patients with abnormal liver function show no obvious symptoms, some progress to severe cholestasis, liver injury, or even liver failure [6–8]. It is clear that there is a complex but intimate bidirectional relationship between thyroid and liver in health and disease [9]. Liver is critical in thyroid hormone activation and inactivation, transport, and metabolism; and liver dysfunction has been consistently reported to affect serum levels of thyroid hormones and their related hormones and autoantibodies [9]. Meanwhile, several studies showed that serum free thyroxine (FT4), thyroid-stimulating hormone (TSH), thyroid-stimulating hormone receptor antibody (TRAb) levels were independent predictors of risk of RAI failure [10–13]. To date, whether liver dysfunction would affect the efficacy of RAI therapy in GD patients has not been well studied. Therefore, to determine the association between liver dysfunction and RAI efficacy, we retrospectively enrolled patients with GD who received RAI therapy, and compared baseline and treatment outcomes at 3-, 6-, and 12-month follow-up between patients with and those without liver dysfunction.

## Materials and methods

### Study patients

The study population consisted of patients with GD who received RAI therapy at the Department of Nuclear Medicine of Nanjing Drum Tower Hospital from January 2013 until December 2016. All patients were diagnosed with GD based on the following criteria [8, 14]: 1) an elevated serum FT4 with undetectable or suppressed serum TSH level; and 2) an elevated serum level of TRAb. Exclusion criteria were as follows: 1) received RAI therapy more than once; 2) lack of follow-up data; 3) had history of thyroid surgery; and 4) had other conditions that could cause liver dysfunction, such as viral hepatitis, alcoholic hepatitis, and autoimmune hepatitis. Liver dysfunction was defined as at least one aminotransferase or bilirubin above normal [15]. The laboratory reference ranges were 5–40 U/L for alanine aminotransferase (ALT), 8–40 U/L for aspartate aminotransferase (AST), 1.7–6.8 μmol/L for direct bilirubin, and 5–20.5 μmol/L for total bilirubin.

### Demographic data, laboratory assays for thyroid function and autoantibodies

Demographic information such as age and sex, and physiologic and clinical data such as weight, disease duration, and heart rate were collected. Serum TSH, free triiodothyronine (FT3), FT4, thyroid autoantibodies (thyroid peroxidase antibody [TPOAb] and thyroglobulin antibody [TgAb]) concentrations were detected by electrochemical luminescence assays with Cobas Eless 601 (Roche). TRAb was measured using a third-generation TBII assay with the automated Cobas electrochemiluminescence immunoassay (Roche). The reference ranges of TSH, TPOAb, TgAb, and TRAb were 0.27–4.2 mIU/L, 0–34 IU/mL, 0–115 IU/mL, and 0–1.75 IU/L respectively [16].

### Thyroid volume, RAI uptake and I131 dose

Thyroid volume (ml) was calculated based on measurements from ultrasound images as length (cm) × width (cm) × height (cm) × 0.479 [17], and thyroid mass was calculated considering a density of 1 g/ml. RAI uptake (2- and 6-h RAIU) was measured. We treated each patient with a dose of 10–15 mCi, a suggested dose for GD [18, 19]. The final treatment dose within the range of 10–15 mCi was decided based on evaluation of clinical symptoms, thyroid mass and radioactive iodine uptake, at the treating physicians’ discretion.

### Follow-ups and outcome measures

We evaluated the response following RAI according to clinical manifestations and laboratory findings. All patients were scheduled to visit the physician and perform clinical and laboratory examinations 3-, 6-, and 12-month after the RAI treatments. Treatment outcomes of RAI were classified according to the recommendations of the 2016 American Thyroid Association (ATA) guidelines for hyperthyroidism and other causes of thyrotoxicosis [4]. RAI therapy was considered effective if there was complete remission, partial remission, or hypothyroidism at 12-month follow-up [20]. Complete remission was defined if patients maintained normal thyroid function without ATDs or levothyroxine and had no symptoms or signs of hyperthyroidism; partial remission was defined if patients showed reduction of serum FT4 and improvement of hyperthyroidism-associated symptoms and signs, both of which however, did not achieve complete normalization [20]. Other outcomes included euthyroidism or hypothyroidism, early-onset hypothyroidism, and recurrence.

### Statistical analysis

Statistical analysis was performed using SPSS v26.0 software (SPSS Inc, Chicago, IL, USA). Data were evaluated with the Kolmogorov–Smirnov test. Variables that conformed to a normal distribution are expressed as means ± standard deviation and were analyzed with the independent samples t test. Variables with a skewed distribution are expressed as median (range) and were analyzed with the rank-sum test. Categorical variables were presented as frequencies (percentages) and compared with the chi-squared test. Binary logistic regression was used to determine risk factors for liver dysfunction and treatment effect. The nonparametric Friedman test was used to evaluate overall changes in indicators at each follow-up time point compared with previous time point. Differences between patients with liver dysfunction and those without were compared at different time-lines. A *p* value < 0.05 was considered statistically significant.

## Results

### Baseline characteristics

**Fig. 1:**
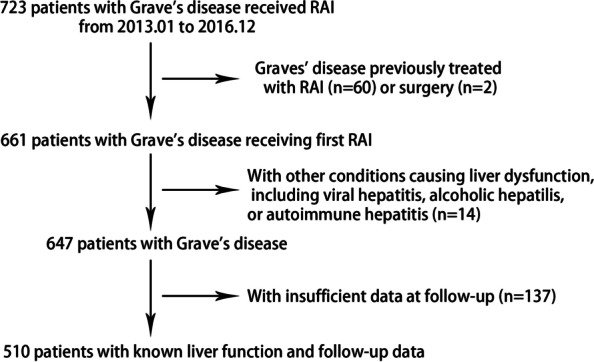
Patient selection process

A total of 723 patients with GD who received RAI were eligible for selection. 213 were excluded and a final 510 cases were included for analysis (Fig. 1). 59 patients (11.6%) received RAI as first-line treatment because of baseline liver dysfunction, leukopenia or patients’ preferences. The other 451 patients (88.4%) received ATD as first-line treatment, but switched to RAI due to drug-induced liver damage, leukopenia, drug allergy, poor medication compliance or unmet efficacy.

Baseline clinical information for the study population is shown in Table 1. Most patients were female (69.4%), and mean age was 41.5 years, mean duration of GD was 4.96 years. Liver dysfunction was recorded in 222 (43.5%) patients, in which 72 patients had moderate to severe liver dysfunction (ALT or AST ≥ 80U/L). Patients with liver dysfunction and normal liver function were similar in terms of age, body weight, heart rate, thyroid volume, 2- and 6-h RAIU, dose of iodine, and serum concentrations of TgAb and TPOAb. Patients with liver dysfunction tended to be male (36.5% vs. 26%, *p* = 0.011), showed a shorter disease duration (3.9 ± 6.0 vs. 5.8 ± 6.6 years, *p* = 0.001), but a higher serum FT3 (median 27.6 vs. 20.6 pmol/L, *p* < 0.001) and FT4 (median 65.4 vs. 53.5 pmol/L, *p* < 0.001) levels, as compared with those with normal liver function (Table 1).Binary logistic regression analysis revealed that duration of disease (OR = 0.951, 95% CI: 0.992–0.980, *p* = 0.001) and male sex (OR = 1.631, 95% CI: 1.116–2.384; *p* = 0.011) were differential factors for liver dysfunction in patients with GD (Table 2).

**Table 1:** Baseline characteristics of patient with Graves’ disease with normal liver function and liver dysfunction

Characteristics	Total	Normal liver function (*n* = 288)	Liver dysfunction (*n* = 222)	*p* value
Male (%)	30.59	26.04	36.49	0.011
Age (years)	41.51 ± 13.85	41.8 ± 14.322	41.13 ± 13.224	0.587
Duration of disease (years)	4.96 ± 6.46	5.8 ± 6.65	3.9 ± 6.0	0.001
Weight (kg)	57.67 ± 9.74	57.05 ± 9.01	58.47 ± 10.59	0.112
Heart rate (bpm)	92.98 ± 16.7	92.84 ± 16.66	93.16 ± 16.81	0.830
Thyroid volume (ml)	21.33 (14.75, 29.91)	22.32 (14.34, 32.19)	20.92 (15.32, 29.19)	0.590
2-h RAIU (%)	59.43 ± 21.44	58.61 ± 21.43	60.48 ± 21.46	0.328
6-h RAIU (%)	78.73 ± 17.46	78.24 ± 17.71	79.36 ± 17.15	0.473
Dose of iodine (mCi)	12.50 ± 3.07	12.59 ± 2.878	12.38 ± 3.304	0.433
TSH (mIU/l)	0.005 (0.005, 0.01)	0.005 (0.005, 0.010)	0.005 (0.005, 0.010)	0.110
FT3 (pmol/l)	22.35 (15.41, 33.20)	20.60 (12.47, 32.78)	27.57 (17.38, 39.45)	< 0.001
FT4 (pmol/l)	60.75 (38.1, 93.79)	53.50 (35.07, 83.35)	65.40 (44.81, 100.00)	< 0.001
TRAb (IU/ml)	14.94 (7.22, 35.48)	12.36 (5.86, 26.95)	14.98 (7.38, 32.77)	0.067
TPOAb (IU/ml)	258.72 (57.32, 565.5)	262.2 (47.3, 596.7)	251.2 (82.5, 501.8)	0.820
TgAb (IU/ml)	201.4 (32.8, 541.1)	190.9 (28.3, 508.3)	206.3 (39.8, 554.0)	0.453
ALT (U/l)	36.2 (24.3, 54.75)	24.00 (17.73, 31.00)	56.55 (45.25, 80.05)	< 0.001
AST (U/l)	28.5 (21.95, 39.7)	22.35 (18.40, 26.68)	40.45 (32.68, 51.33)	< 0.001
ATDs				0.005
None	59	22	37	
PTU	100	60	40	
MMI	351	209	142	

Data are expressed as mean ± standard deviation or median (lower quartile, upper quartile)

*ALT* Alanine aminotransferase, *AST* Aspartate aminotransferase, *ATD* Anti-thyroid drugs, *FT3* Free triiodothyronine, *FT4* Free thyroxine, *MMI* Methimazole, *PTU* Propylthiouracil, *RAIU* Radioactive iodine uptake rate, *TgAb* antithyroglobulin antibody, *TPOAb* Thyroid peroxidase antibody, *TRAb* Thyrotropin receptor antibody, *TSH* Thyroid-stimulating hormone

^*^Statistically significant (*p* < 0.05)

**Table 2:** Binary logistic regression analysis of factors affecting liver function

Indicator	*OR*	95%*CI*		*p* value
		**Lower limit**	**Upper limit**	
Duration of disease	0.951	0.922	0.980	0.001
Male sex	1.631	1.116	2.384	0.011

*CI* Confidence interval, *OR* Odds ratio

^*^Statistically significant (*p* < 0.05)

### Thyroid hormone, autoantibodies and heart rate changes during follow-up after RAI treatment

During follow-up, levothyroxine was being taken by 284 out of the 510 patients at 3 months, by 285 at 6 months, and by 164 patients at 12-month follow-up. Serum FT3, FT4 concentrations decreased whereas TSH level increased significantly at 3 months (all *p* < 0.001). FT3 and FT4 increased between 3 to 6 months (*p* < 0.001) and remained stable thereafter. TSH level did not change significantly after 3 months. Serum levels of TRAb increased in the first 3 months after RAI (*p* = 0.008), remained stable at 3 to 6 months (*p* = 0.629), and decreased significantly after 6 months (*p* < 0.001) (Table 3). Serum TPOAb and TgAb levels showed very similar trend as TRAb during follow-up. Heart rate decreased significantly 3 months post RAI, and remained stable thereafter.

**Table 3:** Overall follow-up indicators at different time points after RAI treatment

Indicators	Baseline	3 months			6 months			12 months			*x*ʹ^*2*^	*P*ʹ
	**Total**	**Total**	***x***^***2***^	***P***	**Total**	***x***^***2***^	***P***	**Total**	***x***^***2***^	***P***		
TSH (mIU/l)	0.005 (0.005, 0.010)	6.650 (0.008, 61.65)	− 1.602	< 0.001	3.800 (0.083, 22.323)	− 0.079	0.425	4.000 (0.760, 13.620)	− 0.022	0.824	404.89	< 0.001
FT3 (pmol/l)	24.95 (14.60, 35.80)	2.96 (1.45, 5.43)	2.257	< 0.001	4.14 (3.27, 5.42)	− 0.543	< 0.001	4.21 (3.66, 4.90)	− 0.030	0.758	600.63	< 0.001
FT4 (pmol/l)	60.29 (38.55, 100.00)	7.42 (3.07, 19.21)	2.178	< 0.001	16.76 (11.58, 21.26)	0.546	< 0.001	16.91 (13.61, 20.43)	− 0.057	0.557	551.69	< 0.001
TRAb (IU/ml)	14.32 (6.99, 32.35)	37.84 (16.53, 40.00)	− 0.942	0.008	25.72 (11.75, 40.00)	− 0.173	0.629	17.52 (5.38, 32.83)	− 1.327	< 0.001	25.055	< 0.001
TPOAb (IU/ml)	295.5 (83.0, 563.8)	600.0 (321.8, 600.0)	− 1.646	< 0.001	537.6 (278.7, 600.0)	− 0.061	0.831	333.6 (128.9, 557.5)	− 1.110	< 0.001	59.049	< 0.001
TgAb (IU/l)	212.3 (39.39, 620.3)	405.1 (120.9, 660.0)	− 0.939	0.001	318.4 (104.7, 650.5)	− 0.415	0.146	149.6 (31.7, 499.5)	− 0.622	0.029	17.692	0.001
Median heart rate (bpm)	93.23 ± 16.53	73.08 ± 12.64	1.438	< 0.001	74.51 ± 13.70	− 0.042	0.911	75.30 ± 12.44	− 0.188	0.615	27.106	< 0.001

Data are expressed as median (lower quartile, upper quartile) unless otherwise indicated

*FT3* Free triiodothyronine, *FT4* Free thyroxine, *TgAb* Antithyroglobulin antibody, *TPOAb* Thyroid peroxidase antibody, *TRAb* Thyrotropin receptor antibody, *TSH* Thyroid-stimulating hormone

^*^Statistically significant (*p* < 0.05)

Follow-up data of GD patients stratified by baseline liver function are shown in Table 4. Serum TSH levels were higher in patients with liver dysfunction at all 3 follow-up time points (*p* = 0.014, 0.008, and 0.025 respectively). FT3 level was lower in patients with liver dysfunction compared to those with normal liver function at 3-month follow-up (*p* = 0.047), but the difference disappeared at 6 and 12 months (*p* = 0.351 and 0.264 respectively). There was no difference in serum levels of FT4, TRAb, TPOAb, and TgAb between the 2 groups at any follow-up time lines (all *p* > 0.05) (Figure S1a–d). Heart rate was lower in patients with liver dysfunction at 3 and 6 months (*p* = 0.017 and 0.026 respectively) but the difference disappeared at 12 months (*p* = 0.177) (Figure S2).

**Table 4:** Follow-up indicators for patients with Graves’ disease with normal liver function and liver dysfunction at different time points after treatment

Indicator	3 months				6 months				12 months			
	**Normal liver function (*n* = 277)**	**Liver dysfunction (*n* = 212)**	**Z/t**	***p* value**	**Normal liver function (*n* = 249)**	**Liver dysfunction (*n* = 184)**	**Z/t**	***P***	**Normal liver function (*n* = 196)**	**Liver dysfunction (*n* = 163)**	**Z/t**	***p* value**
TSH (mIU/l)	1.040 (0.005, 50.270)	16.68 (0.01, 63.90)	− 2.457	0.014	1.580 (0.035, 17.330)	6.315 (0.183, 26.455)	− 2.651	0.008	3.125 (0.262, 9.335)	4.65 (1.230, 16.430)	− 2.242	0.025
FT3 (pmol/l)	3.26 (1.70, 5.78)	2.69 (1.44, 4.80)	1.984	0.047	4.22 (3.38, 5.59)	4.09 (3.23, 5.54)	0.932	0.351	4.27 (3.73, 5.05)	4.14 (3.56, 4.85)	1.117	0.264
FT4 (pmol/l)	9.12 (3.92, 19.37)	6.86 (3.08, 15.09)	1.863	0.062	16.36 (11.46, 21.91)	16.97 (11.69, 21.61)	− 0.212	0.832	16.68 (12.41, 20.59)	17.46 (14.12, 20.42)	− 0.796	0.426
TRAb (IU/ml)	38.02 (24.99, 40.00)	40.00 (16.51, 40.00)	− 0.247	0.805	22.93 (11.42, 40.00)	36.30 (14.13, 40.00)	− 0.969	0.333	12.19 (3.78, 32.12)	21.83 (11.65, 33.68)	− 1.700	0.089
TPOAb (IU/ml)	600.0 (347.4, 600.0)	548.6 (261.5, 600.0)	1.902	0.057	562.7 (226.8, 600.0)	510.7 (277.6, 600.0)	0.119	0.905	336.8 (161.4, 596.8)	319.7 (118.9, 511.2)	1.146	0.252
TgAb (IU/l)	414.2 (194.6, 607.6)	402.9 (160.3, 658.5)	0.363	0.716	400.0 (134.3, 691.2)	310.2 (121.8, 541.3)	1.185	0.236	153.3 (26.62, 524.4)	145.75 (44.10, 463.78)	0.021	0.983
Median heart rate (bpm)	73.97 ± 11.73	70.81 ± 12.11	2.405	0.017	76.86 ± 16.29	72.00 ± 7.87	2.250	0.026	75.43 ± 10.22	75.05 ± 16.05	0.110	0.177

Data are expressed as median (lower quartile, upper quartile) unless otherwise indicated

*FT3* Free triiodothyronine, *FT4* Free thyroxine, *TgAb* Antithyroglobulin antibody, *TPOAb* Thyroid peroxidase antibody, *TRAb* Thyrotropin receptor antibody, *TSH* Thyroid-stimulating hormone

^*^Statistically significant (*p* < 0.05)

### Effect of liver dysfunction on RAI treatment outcome

The efficiency, as defined as complete, partial remission or hypothyroidism following RAI, was comparable between patients with and without liver dysfunction (94.5% vs 90.3%, *p* = 0.142) (Table 5). Also, the incidence differences of early-onset hypothyroidism (87.7% vs 83.4%, *p* = 0.277) and recurrence (4.91% vs 7.14%, *p* = 0.379) were also not statically significant between 2 groups at 12-month follow-up. The rate of normal thyroid function or hypothyroidism was higher in patients with liver dysfunction than in those with normal liver function at 3 months (74.5% vs 62.5%, *p* = 0.005) and 6 months (82.1% vs 69.1%, *p* = 0.002) after RAI treatment, but the difference did not persist at 12-month follow-up (89.6% vs 83.2%, *p* = 0.081) (Table 5). Similar results were found when restricting to patients with moderate to severe liver dysfunction (Table S2). Logistic regression analysis also did not reveal a significant association between liver status and euthyroidism or hypothyroidism at 12-month follow-up (hazard ratio 0.98; 95% CI 0.31 to 3.14, *p* = 0.98) after adjusting for age, FT4, TRAb, thyroid mass, ATD preceding RAI and iodine dose.

**Table 5:** Treatment outcome in patients with Graves’ disease with normal liver function and liver dysfunction at 12-month follow-up

Treatment outcome	Normal liver function (*n* = 196)	Liver dysfunction (*n* = 163)	Total (*n* = 359)	*p* value
Efficiency (n [%])	177 (90.31%)	154 (94.48%)	331 (92.20%)	0.142
Recurrence rate (n [%])	14 (7.14%)	8 (4.91%)	22 (6.13%)	0.379
Euthyroidism or hypothyroidism	163 (83.2%)	146 (89.6%)	309 (86.1%)	0.081
Early-onset hypothyroidism rate (n [%])	164 (83.67%)	143 (87.73%)	307 (85.82%)	0.277

^*^Statistically significant (*p* < 0.05)

## Discussion

To our knowledge, our study remains the first to demonstrate the relationship between baseline liver function with thyroid hormone and RAI outcomes. We found that liver dysfunction was associated with a favorable change in thyroid hormones and TSH following RAI, particularly at short-term follow-up. However, the incidence of efficacy and recurrence were similar between patients with and without liver dysfunction.

The bidirectional interplay between thyroid and liver is intimate and complex in both health and disease status [9]. Liver is the first player in the transport, and metabolism of thyroid hormones. It synthesizes the major thyroid hormone-transport proteins and is critical in regulating circulating thyroid hormone concentrations. Meanwhile, thyroid hormones contribute to hepatocyte metabolic and bilirubin production, partly through modulation of lipid metabolism [9]. Liver dysfunction in GD patients has several potential causes. Excess TH levels can increase cardiac output by 50%–300% in patients with hyperthyroidism compared to healthy individuals, which can lead to chronic congestive heart failure [21]. Liver congestion caused by heart failure can affect liver function. Also, increased splanchnic oxygen consumption and metabolic demands could result in anoxia as well as ischemia in the centro-lobular zones of the liver [22–24]. Additionally, hepatotoxicity occurs in 0.1%–0.2% of patients taking oral medications such as propylthiouracil and methimazole, making it a major side effect of ATDs [25, 26].

RAI therapy is both efficacious and cost-effective in treating GD [27, 28] and is recommended as the first-line treatment by ATA, American Association of Clinical Endocrinologists, and European Thyroid Association guidelines [5, 14]. In patients with GD who develop liver dysfunction after ATD treatment, RAI is a safe alternative that has been shown to improve liver biochemical indices [29, 30].

We found that ALT and AST levels in patients with GD were directly proportional to serum FT3 and FT4 levels, implying that hepatic dysfunction reflects more severe hyperthyroidism. This is supported by the finding that FT4 level was a risk factor for impaired liver function in hyperthyroidism patients [31]. There was no difference in 2- or 6-h RAIU between GD patients with normal liver function and those with liver dysfunction in our study, which is consistent with a previous report that there was no correlation between thyroid iodine uptake rate and liver function in patients with GD [31–33].

Serum levels of FT3 and FT4 declined whereas that of TSH increased after RAI treatment. However, these changes showed a fluctuation at around 6 months post treatment, possibly because RAI therapy was administered with hypothyroidism as the goal, and therefore the ^131^I doses that were used were intended to reduce TH level to near the normal lower limit or even below the reference value as rapidly as possible [4]. TH was gradually restored to a normal and stable level by adjusting the dose of levothyroxine tablets during the follow-up. At 6–12 months post treatment, TSH, FT3, and FT4 levels returned to the normal reference ranges in ~ 85% of the patients. We also observed that levels of thyroid autoantibodies (including TgAb, TPOAb, and TRAb) were elevated after RAI treatment. There are 2 main reasons for this increase—the release of thyroid autoantigen and stimulation of lymphocytes after irradiation. RAI therapy is based on the principle of thyroid follicular cell destruction by beta rays released during ^131^I decay, resulting in reduced TH synthesis and release. However, in this process the damaged thyroid follicular cells release a large amount of thyroid autoantigen that stimulates autoantibody production. Irradiated normal lymphocytes also induce thyroid autoantibody synthesis [34, 35]. In our study, we found that the TRAb levels increased 3 months after RIT, and slightly decreased (but not statistically significant) at 6 months, and significantly decreased at 12 months follow-up. The time course changes in TRAb in our study was largely consistent with that of Fang, which showed an increase of TRAb after 6 months and a decline after 12 months’ RAI [36].

GD patients with abnormal liver function had higher serum TSH levels during the follow-up period compared to those with normal liver function despite there being no differences between the 2 groups at baseline. Moreover, patients with liver dysfunction showed a more rapid decrease in TH level and heart rate in the 3 months after treatment, although TH levels were similar between the 2 groups at 12 months. This difference in FT3 change might be related to a worse thyroid function in hepatic dysfunction group at baseline. We also compared treatment outcomes at 1 year post treatment and found no significant difference in treatment efficacy, recurrence rate, or incidence of early-onset hypothyroidism between groups. Therefore, liver dysfunction seemed to have favorable changes in thyroid hormones and TSH following RAI; however, these favorable changes in patients with liver dysfunction did not translate to better hard outcomes, i.e. less recurrence.

Our study is limited by its retrospective design. Additionally, we evaluated 2- and 6-h RAIU instead because the 24-h measurements were not available in most patients. Prospective studies with a larger sample size are needed to confirm the present findings.

## Conclusion

Liver dysfunction was associated with a favorable change in thyroid hormones and TSH following RAI, particularly at short-term follow-up. However, the incidence of efficacy and recurrence were similar between patients with and without liver dysfunction. Therefore, RAI therapy is safe and effective in treating patients with GD accompanied by liver dysfunction.

## Supplementary Information

**Additional file 1:** **Figure S1.** Serum biochemical indicators (median) stratified by liver function status at different follow-up time points. **Figure S2.** Heart rate (HR, $\overline{\mathrm{x} }$ ± s) stratified by liver function status at different follow-up time points. **Table S1.** Progress of liver function in the overall population and stratified by baseline liver function. **Table S2. **Treatment outcome in patients with Graves’ disease with normal liver function and severe liver dysfunction at 1-year follow-up.

## Data Availability

The datasets generated and/or analysed during the current study are not publicly available due another ongoing study, but are available from the corresponding author on reasonable request.

## References

[1] Taylor PN, Albrecht D, Scholz A, Gutierrez-Buey G, Lazarus JH, Dayan CM, Okosieme OE (2018). Global epidemiology of hyperthyroidism and hypothyroidism. Nat Rev Endocrinol.

[2] Bartalena L, Piantanida E, Gallo D, Ippolito S, Tanda ML (2022). Management of Graves' hyperthyroidism: present and future. Expert Rev Endocrinol Metab.

[3] Liu X, Wong CKH, Chan WWL, Tang EHM, Woo YC, Lam CLK, Lang BHH (2021). Outcomes of Graves' Disease Patients Following Antithyroid Drugs, Radioactive Iodine, or Thyroidectomy as the First-line Treatment. Ann Surg.

[4] Ross DS, Burch HB, Cooper DS, Greenlee MC, Laurberg P, Maia AL, Rivkees SA, Samuels M, Sosa JA, Stan MN (2016). 2016 American Thyroid Association Guidelines for Diagnosis and Management of Hyperthyroidism and Other Causes of Thyrotoxicosis. Thyroid.

[5] Franklyn JA, Boelaert K (2012). Thyrotoxicosis. The Lancet.

[6] Yang J, Li LF, Xu Q, Zhang J, Weng WW, Zhu YJ, Dong MJ (2015). Analysis of 90 cases of antithyroid drug-induced severe hepatotoxicity over 13 years in China. Thyroid.

[7] Regelmann MO, Miloh T, Arnon R, Morotti R, Kerkar N, Rapaport R (2012). Graves' disease presenting with severe cholestasis. Thyroid.

[8] Bartalena L (2013). Diagnosis and management of Graves disease: a global overview. Nat Rev Endocrinol.

[9] Piantanida E, Ippolito S, Gallo D, Masiello E, Premoli P, Cusini C, Rosetti S, Sabatino J, Segato S, Trimarchi F (2020). The interplay between thyroid and liver: implications for clinical practice. J Endocrinol Invest.

[10] Finessi M, Bisceglia A, Passera R, Rossetto Giaccherino R, Pagano L, Castellano G, Ghigo E, Bisi G, Deandreis D (2021). Predictive factors of a worse response to radioactive Iodine-I131 treatment in hyperthyroidism: outcome analysis in 424 patients. A single centre experience Endocrine.

[11] Kim MJ, Cho SW, Kim YA, Choi HS, Park YJ, Park DJ, Cho BY (2022). Clinical Outcomes of Repeated Radioactive Iodine Therapy for Graves' Disease. Endocrinol Metab (Seoul).

[12] Shalaby M, Hadedeya D, Toraih EA, Razavi MA, Lee GS, Hussein MH, Weidenhaft MC, Serou MJ, Ibraheem K, Abdelgawad M (2022). Predictive factors of radioiodine therapy failure in Graves' Disease: A meta-analysis. Am J Surg.

[13] Verdickt S, Van Nes F, Moyson C, Maes T, Van Crombrugge P, Van den Bruel A, Decallonne B. TPO antibody status prior to first radioactive iodine therapy as a predictive parameter for hypothyroidism in Graves' disease. Eur Thyroid J. 2022;11(4):e220047. 10.1530/ETJ-22-0047 PMC9254270 35687484

[14] Kahaly GJ, Bartalena L, Hegedus L, Leenhardt L, Poppe K, Pearce SH (2018). 2018 European Thyroid Association Guideline for the Management of Graves' Hyperthyroidism. Eur Thyroid J.

[15] Wolf PL (1999). Biochemical diagnosis of liver disease. Indian J Clin Biochem.

[16] Zhang X, Ze Y, Sang J, Shi X, Bi Y, Shen S, Zhang X, Zhu D: Risk factors and Diagnostic Prediction Models for Papillary Thyroid Carcinoma. Front Endocrinol. 2022. 10.3389/fendo.2022.938008 PMC9483149 36133306

[17] Perry RJ, Hollman AS, Wood AM, Donaldson MDC (2002). Ultrasound of the thyroid gland in the newborn: normative data. Arch Dis Child Fetal Neonatal Ed.

[18] Bahn Chair RS, Burch HB, Cooper DS, Garber JR, Greenlee MC, Klein I, Laurberg P, McDougall IR, Montori VM, Rivkees SA (2011). Hyperthyroidism and other causes of thyrotoxicosis: management guidelines of the American Thyroid Association and American Association of Clinical Endocrinologists. Thyroid.

[19] De Leo S, Lee SY, Braverman LE (2016). Hyperthyroidism Lancet.

[20] Clinical guidelines for 131I treatment of Graves’ hyperthyroidism (2021 edition). Chin J Nuel Med Mol Imaging. 2021;41(4):242–53.

[21] Klein I, Danzi S (2007). Thyroid disease and the heart. Circulation.

[22] Shimizu Y (2008). Liver in systemic disease. World J Gastroenterol.

[23] Myers JD, Brannon ES, Holland BC (1950). A correlative study of the cardiac output and the hepatic circulation in hyperthyroidism. J Clin Invest.

[24] Malespin M, Nassri A (2019). Endocrine Diseases and the Liver: An Update. Clin Liver Dis.

[25] Cooper DS (1998). Antithyroid drugs for the treatment of hyperthyroidism caused by Graves' disease. Endocrinol Metab Clin North Am.

[26] Cooper DS (2005). Antithyroid drugs. N Engl J Med.

[27] De Leo S, Lee SY, Braverman LE (2016). Hyperthyroidism. The Lancet.

[28] Patel NN, Abraham P, Buscombe J, Vanderpump MP (2006). The cost effectiveness of treatment modalities for thyrotoxicosis in a U.K. center. Thyroid.

[29] Ding Y, Xing J, Qiu Z, Wang Y, Zhang Y, Fang Y, Peng X, Long Y, Deng P (2016). Radioactive Iodine Therapy without Recent Antithyroid Drug Pretreatment for Hyperthyroidism Complicated by Severe Hyperbilirubinemia Due to Hepatic Dysfunction: Experience of a Chinese Medical Center. Endocr Pract.

[30] Chawla M, Bai CS (2008). Four Cases of Coexistent Thyrotoxicosis and Jaundice: Results of Radioiodine Treatment and a Brief Review. Thyroid.

[31] Li C, Tan J, Zhang G, Meng Z, Wang R, Li W, Zheng W (2015). Risk factors of hyperthyroidism with hepatic function injury: a 4-year retrospective study. Horm Metab Res.

[32] Malik R, Habib M, Tootle R, Hodgson H (2005). Exogenous thyroid hormone induces liver enlargement, whilst maintaining regenerative potential–a study relevant to donor preconditioning. Am J Transplant.

[33] Wang R, Tan J, Zhang G, Zheng W, Li C (2017). Risk factors of hepatic dysfunction in patients with Graves' hyperthyroidism and the efficacy of 131iodine treatment. Medicine (Baltimore).

[34] Chiovato L, Fiore E, Vitti P, Rocchi R, Rago T, Dokic D, Latrofa F, Mammoli C, Lippi F, Ceccarelli C (1998). Outcome of thyroid function in Graves' patients treated with radioiodine: role of thyroid-stimulating and thyrotropin-blocking antibodies and of radioiodine-induced thyroid damage. J Clin Endocrinol Metab.

[35] McGregor AM, McLachlan SM, Smith BR, Hall R (1979). Effect of irradiation on thyroid-autoantibody production. Lancet.

[36] Fang Y, Du WH, Zhang CX, Zhao SX, Song HD, Gao GQ, Dong M (2021). The effect of radioiodine treatment on the characteristics of TRAb in Graves' disease. BMC Endocr Disord.

