# Supplementary material for: Effect of liver dysfunction on outcome of radioactive iodine therapy for Graves’ disease
Source: BMC Endocr Disord. 2022 Dec 15;22:319. doi: 10.1186/s12902-022-01242-w (PMC9753361; doi:10.1186/s12902-022-01242-w)
Supplement: Supplementary file 1 — Additional file 1: Figure S1. Serum biochemical indicators (median) stratified by liver function status at different follow-up time points. Figure S2. Heart rate (HR, $\overline{\mathrm{x} }$x¯ ± s) stratified by liver function status at different follow-up time points. Table S1. Progress of liver function in the overall population and stratified by baseline liver function. Table S2. Treatment outcome in patients with Graves’ disease with normal liver function and severe liver dysfunction at 1-year follow-up. [file 12902_2022_1242_MOESM1_ESM.docx]

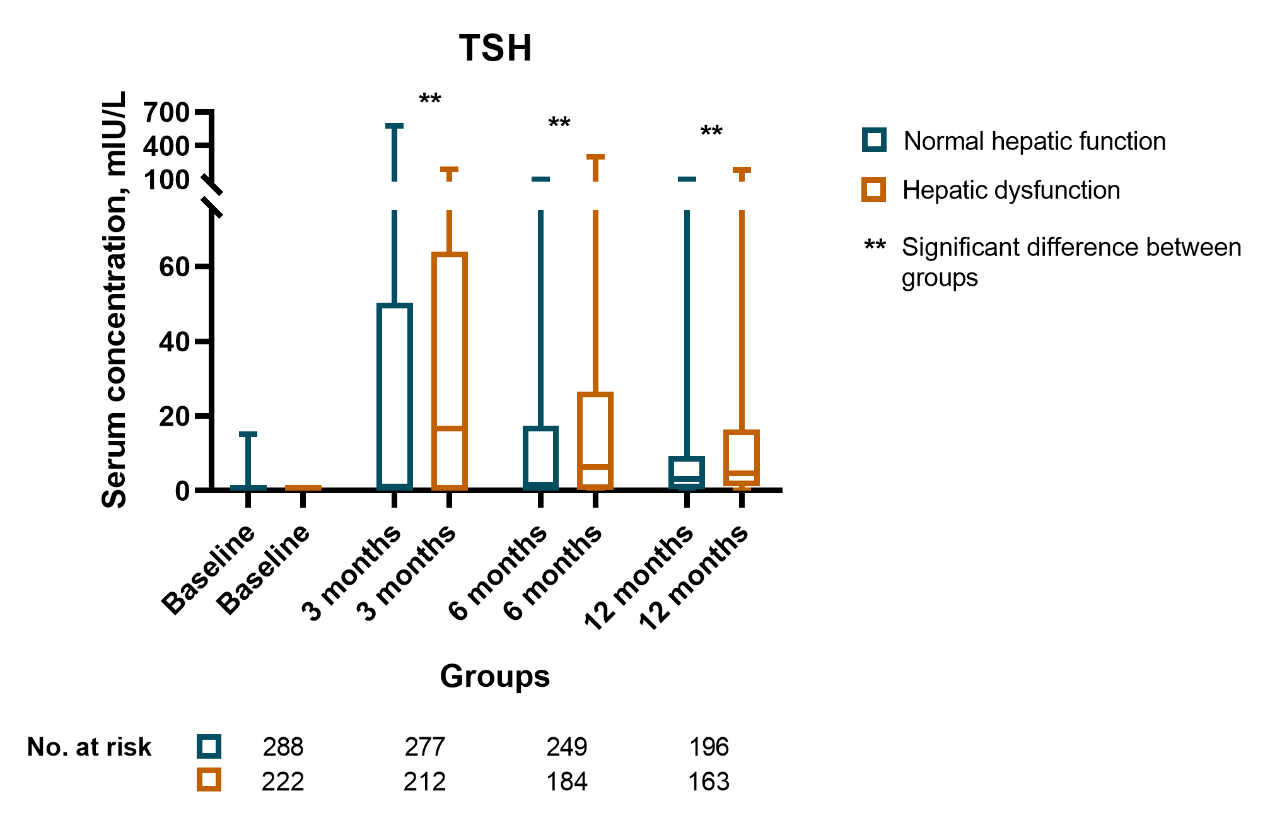


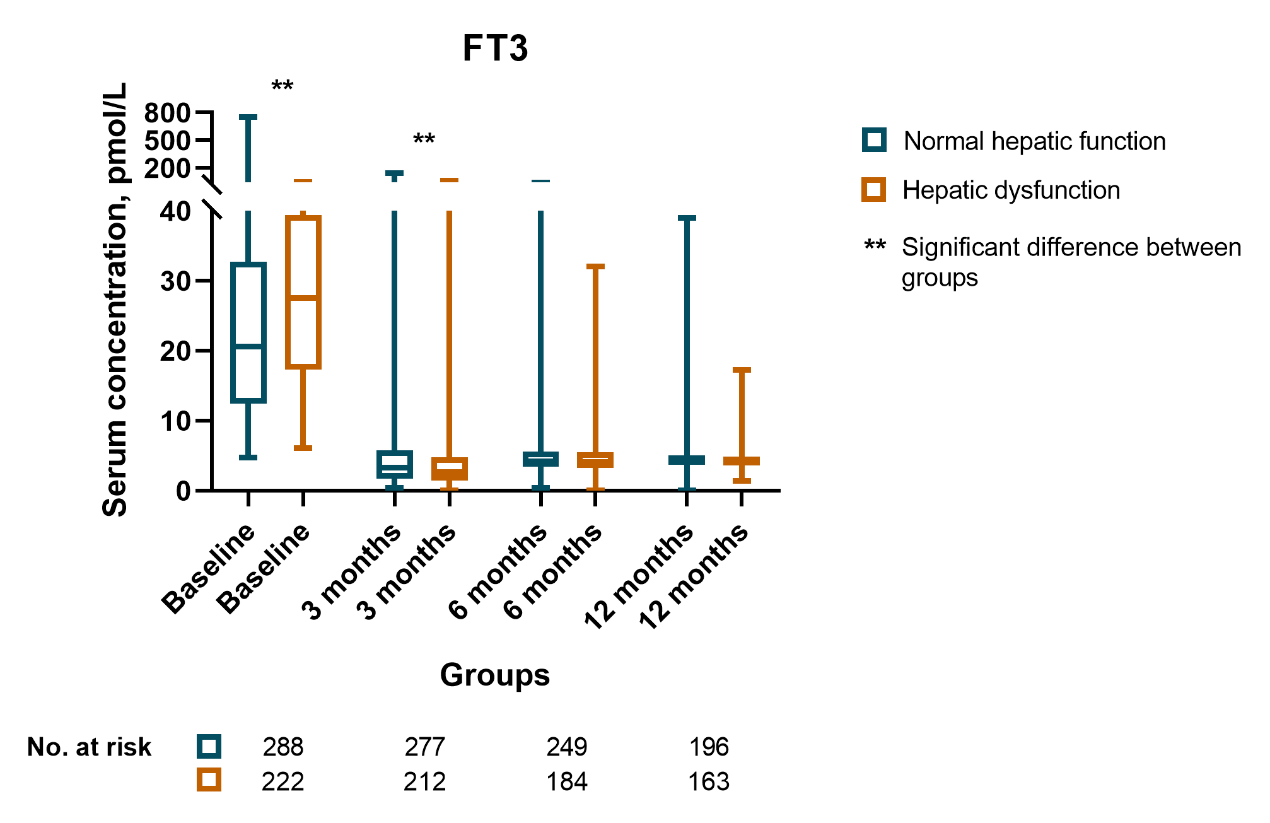


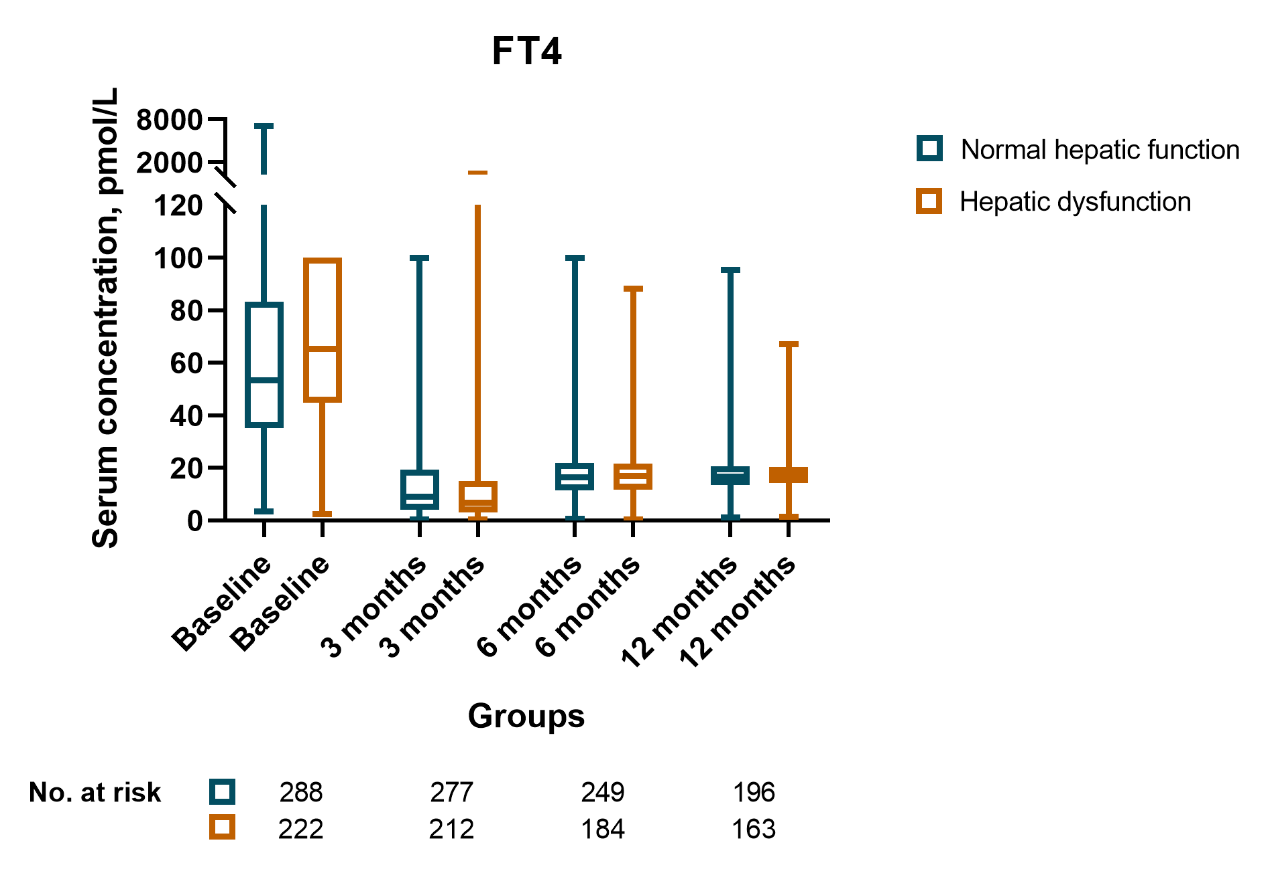


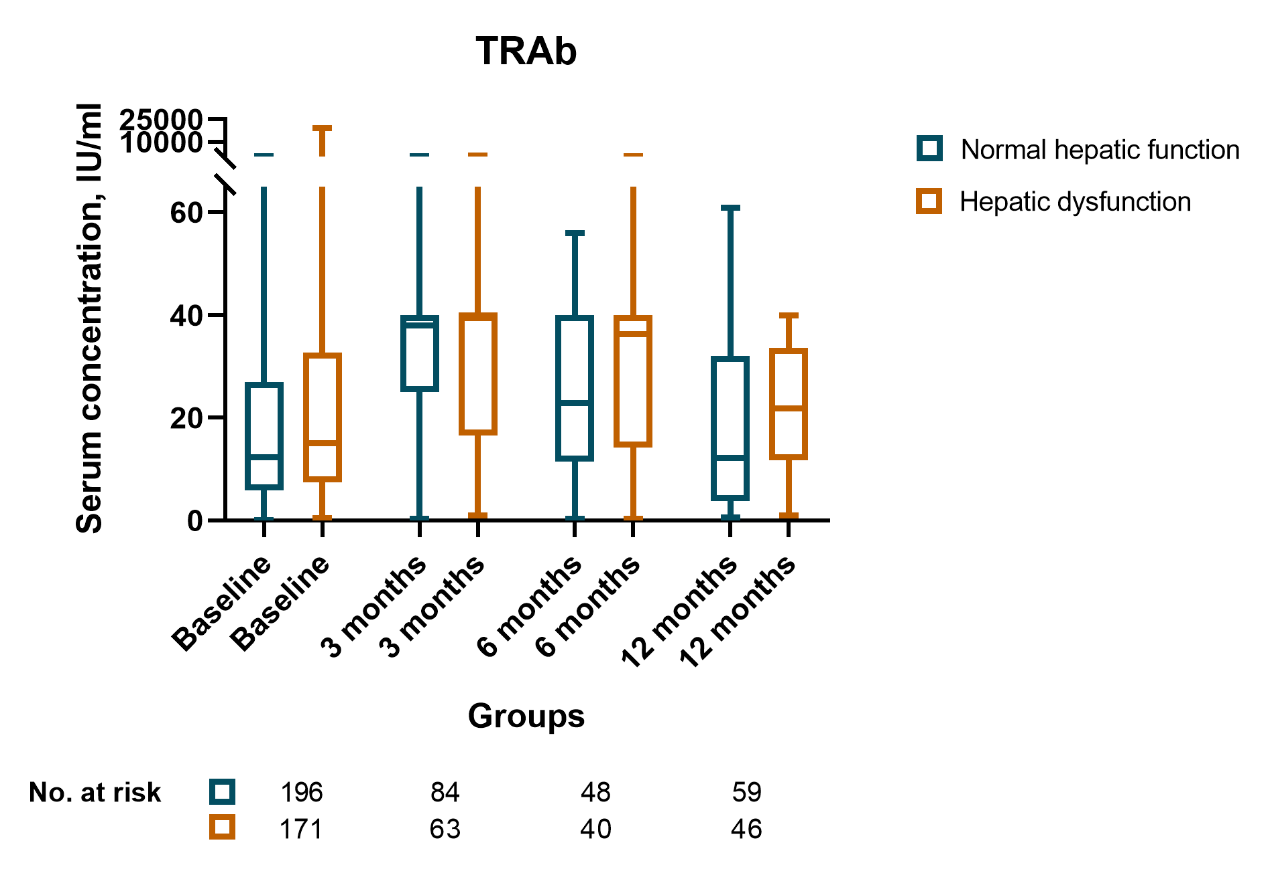


**Figure S1.** Serum biochemical indicators (median) stratified by liver function status at different follow-up time points.

**Figure S2.** Heart rate (HR, $\bar{x}$±s) stratified by liver function status at different follow-up time points.

**Table S1.** Progress of liver function in the overall population and stratified by baseline liver function

| **ALT (U/l)** | **N** | **Baseline** | **3 months** | | |
| --- | --- | --- | --- | --- | --- |
|  |  | **Total** | **Total** | ***Z*** | ***P*** |
| Total | 101 | 47.0 (35.15, 72.15) | 33.9 (21.75, 49.30) | −4.158 | <0.001 |
| Normal liver function | 23 | 26.8 (18.00, 35.10) | 26.3 (18.10, 43.10) | −1.597 | 0.110 |
| Liver dysfunction | 78 | 51.0 (44.00, 82.35) | 34.1 (23.00, 49.70) | −4.764 | <0.001 |

| **AST (U/l )** | **N** | **Baseline** | **3 months** | | |
| --- | --- | --- | --- | --- | --- |
|  |  | **Total** | **Total** | ***Z*** | ***P*** |
| Total | 101 | 37.1 (26.15, 48.25) | 33.7 (25.05, 45.65) | −0.502 | 0.616 |
| Normal liver function | 23 | 23.1 (17.60, 26.60) | 26.3 (18.10, 43.10) | −3.680 | <0.001 |
| Liver dysfunction | 78 | 42.2 (33.43, 51.88) | 33.7 (25.25, 45.43) | −2.122 | 0.034 |

**Table S2.** Treatment outcome in patients with Graves’ disease with normal liver function and severe liver dysfunction at 1-year follow-up.

| **Treatment outcome** | **Normal liver function (n=196)** | **Moderate to severe liver dysfunction (n=65)** | ***P value*** |
| --- | --- | --- | --- |
| Efficiency (n [%]) | 177 (90.31%) | 61 (93.38%) | 0.459 |
| Recurrence rate (n [%]) | 14 (7.14%) | 5 (7.69%) | 0.99 |
| Euthyroidism or hypothyroidism | 163 (83.2%) | 53 (81.54%) | 0.764 |
| Early-onset hypothyroidism rate (n [%]) | 164 (83.67%) | 51 (78.5%) | 0.339 |
